# Midwifery Care: An Evolutionary Concept Analysis

**DOI:** 10.1002/nop2.70354

**Published:** 2025-11-09

**Authors:** Martina Barbieri, Andrea Moro, Gianluca Catania, Franco A. Carnevale, Giuseppe Aleo, Milko Zanini, Loredana Sasso, Annamaria Bagnasco

**Affiliations:** ^1^ Department of Health Sciences University of Genoa Genoa Italy; ^2^ Nursing Office IEO European Institute of Oncology IRCCS Milan Italy; ^3^ Ingram School of Nursing McGill University Montreal Quebec Canada; ^4^ Faculty of Nursing and Midwifery Royal College of Surgeons in Ireland Dublin Ireland

**Keywords:** concept analysis, family‐centred care, midwifery care, relationship, safety

## Abstract

**Aim:**

To build an evidence‐based definition of midwifery care and its fundamental and distinctive features.

**Design:**

Rodgers and Knafl's evolutionary concept analysis.

**Review Methods:**

Six databases (PubMed, CINAHL, Web of Science, PsycINFO, Scopus, ProQuest) were searched and a thematic sampling of the sources was performed.

**Data Sources:**

The search yielded 30 relevant papers.

**Results:**

Key findings include five antecedent categories: philosophy, personal features, regulatory features, care context and professional team. Attributes include relationship and family‐centredness. Consequences encompass safety, empowerment and professional outcomes. Related concepts and surrogate terms reflect the broader scope and the fragmented perception of ‘midwifery care’.

**Discussion:**

Midwifery care is often limited by obstetric‐led models that prioritise risk management over holistic care. This disparity leads to discrimination and professional dissatisfaction, impacting on the quality of care and midwives' well‐being.

**Implications for the Profession:**

Through the conceptualisation of midwifery care, research, education, clinical practice and governance can be oriented toward professional priorities, enhancing coherence, awareness and relevance in relation to the ontological nature of the profession and its systemic value within healthcare and society. In this context, it is crucial for policymakers to maximise the development and implementation of policies that support the establishment of care models based on the principles of midwifery care, ensuring a more comprehensive and effective healthcare system.

**Patient or Public Contribution:**

No patient or public contribution.

## 1 Introduction

The recent recognition of midwifery knowledge, skills and practices as Intangible Cultural Heritage of Humanity by UNESCO (2023) underscores the international relevance of Midwifery Care (MC) within health systems. Global health authorities widely acknowledge the central role of midwives in promoting the health of women, newborns, families and communities (United Nations Population Fund ICoM, World Health Organization 2021).

By supporting the physiological course of pregnancy and birth, midwives contribute to reducing complications and minimising the need for advanced medical interventions (Kennedy et al. 2018). Furthermore, investment in midwifery is recognised as a key strategy for preventing abuse and disrespect in healthcare, particularly among marginalised and vulnerable groups (United Nations Population Fund ICoM, World Health Organization 2021).

However, despite its global relevance, a shared definition of MC is still lacking, which limits the ability to recognise its scope, ensure its quality and distinguish it from overlapping roles within healthcare systems (Renfrew et al. 2014).

## 2 Background

The scope of practice of midwives delineates the activities, tasks and responsibilities that appropriately trained and educated midwives are expected to carry out within health systems globally (International Confederation of Midwives 2023). Despite strong international recommendations, the full implementation of this scope remains challenging in many healthcare contexts (Watkins et al. 2023). Consequently, midwives frequently report professional dissatisfaction, perceiving themselves as mediators operating in environments misaligned with the philosophy underpinning MC (Watkins et al. 2023). Moreover, MC is often insufficiently acknowledged, with its distinctive approach and specific characteristics inadequately valued (McFarland et al. 2020).

In contrast, when midwives are fully recognised and enabled to provide high‐quality MC, they demonstrate greater motivation to remain in the profession (Bloxsome et al. 2020). Strengthening MC also appears essential to counterbalance the dominant influence of medicalized models of care, which often undermine midwives' autonomy and diminish the value of their contributions in clinical settings (McFarland et al. 2020).

Although the literature provides consistent insights into midwives' competencies (International Confederation of Midwives 2019), attitudes (International Confederation of Midwives 2014), and professional roles (International Confederation of Midwives 2023), there remains an absence of a conceptual definition of MC as a distinct care approach. While the need for a clear definition has been previously recognised as a prerequisite for ensuring care quality, it has not yet been adequately addressed (Renfrew et al. 2014).

Accordingly, the aim of this concept analysis is to develop an evidence‐based definition of MC and to identify its fundamental and distinguishing attributes. In addition, this analysis seeks to explore the themes emerging from the literature that influence the characteristics, implementation and delivery of MC.

## 3 Methods

This study is an evolutionary concept analysis (Rodgers and Knafl 2000). This method was selected because it serves the aim of this study, enabling to clarify the meanings of broad concepts, allowing for precision in investigations and the development of discipline‐specific knowledge. By conducting a concept analysis, the existing characteristics of MC can be reviewed and synthesised, offering a valuable opportunity to draw insights from scientific publications and propose a comprehensive clarification of the concept (Parse 2022).

Table 1 presents the definitions of the concept's key elements as identified through the analysis, in accordance with Rodgers' evolutionary methodology (Rodgers and Knafl 2000).

**TABLE 1:** Definitions of the conceptual elements identified according to Rodgers' evolutionary methodology.

Elements	Definitions
Antecedents	Contextual or situational factors that must be present prior to the occurrence of the concept.
Attributes	Core characteristics or features that consistently define the concept and distinguish it from others.
Consequences	Outcomes or results that occur as a result of the concept being present or implemented.
Surrogated terms	Terms that are commonly used as synonyms of the concept even though they do not have exactly the same meaning.
Related concepts	Concepts that are somehow related to the concept under analysis, even if they do not share the same group of attributes, antecedents and consequences.

Specifically, Rodgers' evolutionary concept analysis is particularly suited for MC because it views concepts as dynamic and evolving, shaped by context and time. Rooted in structuralist and hermeneutic traditions, this approach recognises that concepts are abstractions that develop through their use and significance in practice.

The cyclical process of concept development, which involves continuous application and redefinition, aligns with the changing nature of midwifery, where concepts must adapt to evolving healthcare practices and patient needs. This method allows for an inductive clarification of concepts based on their real‐world use, making it ideal for a practice‐oriented field like midwifery.

According to the chosen approach, the six activities of Rodgers' evolutionary concept analysis were undertaken as shown in Table 2.

**TABLE 2:** Rodgers' steps of evolutionary analysis of the concept of midwifery care.

	Activities involved in Rodgers' method	Concept analysis of midwifery care
1	Identify the concept of interest and associated expressions	Concept: midwifery care Surrogated terms: maternity, maternity care Related concepts: sexual health care, reproductive health care
2	Identify and select the appropriate realm (setting and sample) for data collection	No time limits imposed Peer‐reviewed journal articles
3	Collect data relevant to the attributes and the contextual basis of the concept	Inductive analysis to collect and analyse raw data (attributes, antecedents, consequences, surrogated terms, common themes)
4	Analyse data regarding the above characteristic of the concept	Thematic analysis to categorise raw data
5	Identify an exemplar of the concept, if appropriate	Identified
6	Identify implications, hypothesis and implications for further development of the concept	Attributes, antecedents, consequences and surrogated terms discussed in view of the common themes identified

## 4 Data Sources

Firstly, researchers identified the inclusion criteria, keywords and the search strategy as shown in Table 3.

**TABLE 3:** Search strategy.

Inclusion criteria	Keywords and string
Studies with as main aim the one to describe, analyse or theorise midwifery care Studies with as main aim the one to describe, analyse or theorise the role of midwives Studies with as main aim the one to describe, analyse or theorise specific aspects of midwifery care Studies with as main aim the one to describe, analyse or theorise organisational models or care delivery models in which midwifery care is delivered Studies were considered eligible regardless of the context, study design, population or setting English or Italian language No time limits	(Midwifery OR ‘Midwifery Care’) AND (Maternity OR ‘Maternity Care’ OR ‘Sexual Health Care’ OR ‘Reproductive Health Care’) AND (Theory OR ‘Theoretical framework’ OR ‘Conceptual Framework’ OR ‘Model of Care’ OR ‘Care Delivery Model’ OR ‘Provision of Care’ OR Anthropology OR Sociology OR Psychology)

It is worthy to note that at this early stage of the study the researchers identified *Maternity*, and *Maternity Care*, as surrogated terms.

Furthermore, *Sexual Health Care* and *Reproductive Health Care* were identified as related concepts. In consideration of their relevance related to the concept, all these terms were included to broaden the search strategy.

## 5 Analysis of the Sources

Each included source was analysed independently by two researchers (AM and MB). Initially, a thematic analysis was performed to identify themes corresponding to the categories of antecedents, attributes and consequences related to MC.

During this phase, researchers also identified surrogate terms and related concepts within the sources. Moreover, the initial analysis involved noting common themes across sources that could contribute to a comprehensive understanding of MC.

In the subsequent phase, the raw themes were synthesised to form categories that encompassed multiple conceptually related themes. These categories were then analysed to elucidate the antecedents, attributes, consequences, surrogate terms and related concepts of MC. Based on these categories, a comprehensive definition of MC was developed. Finally, the coded data were reviewed by the research team to ensure consistency and agreement.

## 6 Findings

### 6.1 Search Results

The search strategy as used to query 6 databases for a total of 2171 records, from which 996 duplicates were removed, leaving us with 1175 records. Two researchers (AM and MB) screened the titles and abstracts and then the full texts using the Rayyan website (Ouzzani et al. 2016).

**FIGURE 1:**
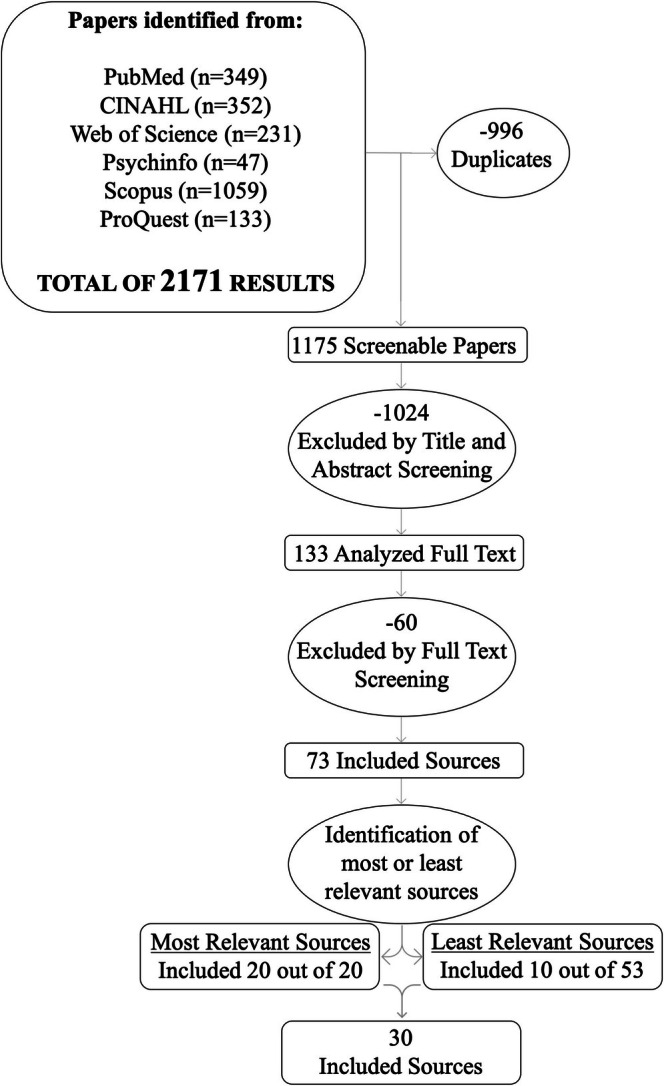
Flow diagram of the screening process.

The screening process is shown in Figure 1. At the end of these two phases any conflict between the two researchers was solved by reaching consensus. The total number of included papers was 73.

During the full‐text screening, the researchers were able to evaluate the relevance of each source in relation to the study's objectives. This process highlighted a spectrum of the relevance of the included sources, reflecting variations in relation to the predetermined inclusion criteria.

To ensure a systematic categorisation, each source was classified according to these criteria, and thematically consistent papers were organised into four groups. The most relevant sources were those with a primary focus on ‘Midwifery Care’ (*N* = 17) and ‘Midwives’ (*N* = 3), while sources classified as less relevant focused on ‘Specific Aspects of Midwifery Care’ (*N* = 21) and ‘Organizational Models or Care Delivery Models’ (*N* = 33).

In line with established methodology (Rodgers and Knafl 2000), which recommends the inclusion of at least 30 sources, we adopted a two‐step selection process.

First, all the highly relevant sources were included. To meet the required minimum number of sources, we then selected additional papers from the less relevant categories, prioritising the most recent publications.

This selection was again guided by the study's aim to capture contemporary conceptualisations of MC. Specifically, 3 papers from the group ‘Specific Aspects of Midwifery Care’ and 7 papers from the ‘Organizational Models or Care Delivery Models’ group were included.

Table 4 reports the details about the included sources.

**TABLE 4:** Author, year, country, title, aim, methods, and results of included sources.

Authors, year, country	Title	Aim	Methods	Results
Sharp (1998), USA	Ethics in reproductive health care: A midwifery perspective	To explore the emerging ethical issues in screening, diagnostic, and treatment options for women of reproductive age, examining the challenges and pressures faced by women, healthcare professionals, and society.	Analytical exploration	Threats to the effectiveness of midwifery practice are identified, analyzing the instrumental and expressive roles of midwives, and solutions are proposed to address ethical dilemmas, highlighting the distinction between midwifery practice at the micro level and policy decisions at the macro level.
Fahy and Parratt (2006), Australia	Birth Territory: A theory for midwifery practice	To introduce the theory of ‘Birth Territory’	Inductive theory synthesis	The theory is applied to two distinct clinical stories occurring in a hospital setting, demonstrating that when midwives effectively use ‘midwifery guardianship’ to create an optimal Birth Territory, women are more likely to experience natural births, increased satisfaction, and smoother post‐birth adjustments, while also benefiting the baby and positively impacting families and society.
Byrom and Downe (2010), UK	‘She sort of shines’: midwives' accounts of ‘good’ midwifery and ‘good’ leadership.	To examine the personal perception of participants about midwifery and leadership	Phenomenological interview survey with thematic analysis	The study identified two main dimensions of ‘good’ leadership and midwifery: skilled competence, emphasizing knowledge and safety, and emotional intelligence, highlighting the importance of emotional capability in both midwives and leaders.
Walsh and Devane (2012), UK	A metasynthesis of midwife‐led care.	To investigate the reason why midwife‐led care are associated with less intervention rates with no associated increase harm	Metasynthesis	Overall, lower intervention rates were associated with the increased agency of both women and midwives, with the smaller scale of care settings contributing to these outcomes.
Page and Mander (2014), UK	Intrapartum uncertainty: A feature of normal birth, as experienced by midwives in Scotland.	To explore how midwives perceive uncertainty when caring for women in low risk labor	Grounded Theory, unstructured in‐depth one‐to‐one interviews and focus groups	The analysis revealed three categories: intrapartum uncertainty, normality boundaries, and threshold pressures. Midwives define normal labor based on their tolerance for uncertainty, with supportive environments helping them maintain this normality. Those who can tolerate uncertainty are more likely to view labor as normal, while external pressures can either expand or contract their definitions of what is considered normal.
Brown et al. (2016), Australia	Cultural safety and midwifery care for Aboriginal women—A phenomenological study.	To explore the experiences of midwives when providing cultural care, and to investigate their understandings of cultural safety in their practice	Interpretive Heideggerian phenomenology, semi‐structured interviews	Six main themes emerged: (1) connecting with women; (2) building support networks through cultural knowledge; (3) overcoming barriers to effective care; (4) equating perceived equity with uniform treatment; (5) understanding culture; and (6) assessing urban versus rural Aboriginal cultural needs. Midwives highlighted the importance of communication and collaboration with
Meier Magistretti et al. (2016), Switzerland	Setting the stage for health: Salutogenesis in midwifery professional knowledge in three European countries	To investigate if midwives' conducts in health‐oriented settings were consistent with the orientation itself	Narrative interviews. In‐depth and comparative pattern data analyses were performed.	Three main concepts emerged, illustrating the midwives' practice orientation rooted in Antonovsky's salutogenic theory (comprehensibility, manageability, and meaningfulness). The midwives' implicit knowledge promoted health‐enhancing experiences for families during maternity care, suggesting that a health orientation can be fostered in practice by examining midwives' experiences in supportive environments.
Hunter et al. (2016), New Zeland	Generosity of spirit sustains caseloading Lead Maternity Carer midwives in New Zealand.	To investigate what sustains midwives who have worked in the LMC model of midwifery care for more than 8 years	Qualitative descriptive study, interviews	The analysis identified key themes emphasizing that the generosity of spirit among midwives, when aligned with established personal and professional boundaries, is essential for sustaining caseload practice. This synergy enhances reciprocity and partnership, contributing to the joy of practice and ensuring the sustainability of LMC midwifery over time.
Borrelli et al. (2016), UK, Australia	The kaleidoscopic midwife: A conceptual metaphor illustrating first‐time mothers' perspectives of a good midwife during childbirth. A grounded theory study	To explore first‐time mothers' expectations and experiences of a good midwife in the context of labor and birth care	Qualitative Straussian grounded theory, semi‐structured interviews	The study introduced the dynamic model of ‘The kaleidoscopic midwife,’ reflecting first‐time mothers' views on a good midwife. This model highlights four pillars of intrapartum care: promoting individuality, supporting embodied limbo, helping to go with the flow, and providing information and guidance. The kaleidoscopic midwife adapts to each woman's unique needs, fostering an environment that helps her navigate labor's uncertainties through relationship‐mediated support, knowledgeable actions, physical presence, and immediate availability.
Newton et al. (2016), Australia	Understanding the ‘work’ of caseload midwives: A mixed‐methods exploration of two caseload midwifery models in Victoria, Australia	To explore caseload and standard care midwives' views and experiences in two new caseload models in Victoria, Australia.	Mixed‐methods: Quantitative data via two cross‐sectional surveys; qualitative data from in‐depth interviews	Identified two themes: (1) Caseload midwifery is a distinct work model, involving on‐call duties and balancing personal/work time to prevent burnout; (2) Caseload midwives view their role as ‘real’ midwifery, emphasizing relationship‐building, responsibility, autonomy, and legitimacy.
Wallace (2019), USA	Using a Birth Center Model of Care to Improve Reproductive Outcomes in Informal Settlements‐a Case Study.	To present a model of service delivery, which could be sustainable and acceptable for marginalized women living in informal settlements	Description of three case studies	Birth centers can provide high‐quality, respectful, and culturally appropriate care, improving maternal health outcomes in informal settlements compared to current conditions.
Dettwyler et al. (2019), USA	Certified Nurse‐Midwives' Experiences With Provision of Prenatal Genetic Screening: A Case for Interprofessional Collaboration	To explore the experiences of Midwives with the prenatal genetic screening provision	Grounded Theroy, semi‐structured interviews	Six themes related to CNMs' prenatal GS provision were identified: clinical protocols, patient education, shared decision‐making, testing initiation, results delivery, and follow‐up coordination. NIPT was found to align with midwifery's noninterventionist philosophy, though CNMs reported limited experience with it.
Coates and Foureur (2019), Australia	The role and competence of midwives in supporting women with mental health concerns during the perinatal period: A scoping review	To map the literature relevant to a broad research question or topic to gain insight into the nature of the evidence and identify research gaps	Scoping review	Findings indicate midwives are interested in providing mental health support but lack confidence, knowledge, and training. Appropriate training and organizational support can address these deficits. There is evidence that midwife‐led counseling interventions are effective.
Eri et al. (2020), Norway, Sweden, Denmark, Iceland	Models for midwifery care: A mapping review.	To gain an overview about published papers which focus on theoretical frameworks for Midwifery Care	Mapping review	Six models from six studies were included, developed through different methodologies and philosophical underpinnings. Common characteristics included emphasis on the midwife‐woman relationship, woman‐centredness, and a salutogenic focus in care.
Niles et al. (2021), USA	Kairos care in a Chronos world: Midwifery care as model of resistance and accountability in public health settings.	To explore how midwives perceive their own role while caring for minoritized communities	In‐depth, semi‐structured interviews	The central theme “Kairos care in a Chronos World” illustrates the delivery of individualized, health‐promoting care amidst a system focused on measurement and efficiency. Five subthemes emerged: (1) the politics of progress, (2) normalizing pathologies, (3) cherished connections, (4) protecting the experience, and (5) caring for the social body.
Wikberg (2021), Finland	A theory on intercultural caring in maternity care	To describe a theory on intercultural caring in maternity care and its development	Qualitative methodology using hermeneutics to interpret previous studies.	Five key themes emerged: (1) the relationship between caring and power; (2) the constant presence of family; (3) multiple vulnerabilities related to childbearing and migration; (4) the transformative effect of intercultural encounters on both mother and midwife; (5) conflicts as catalysts for change. The theory includes four dimensions of intercultural caring: universal, cultural, contextual, and unique, emphasizing the impact of external circumstances on maternity care.
Naughton et al. (2021), Australia	Providing woman‐centred care in complex pregnancy situations.	To better understand the concept of women centred care in the context of complex pregnancies	Integrative review	Organizational and professional power differentials create barriers to woman‐centred care, leading to professional boundary tensions and increasing the risk of women with complex pregnancies ‘falling through the gaps’ in maternity services.
Converso et al. (2021), Italy	Born in translation: Midwifery practice with pregnant migrants—Between stereotypes and empathy	To explore the professional's experience in coping with different maternity habits, their interpretation of migrants' representations and needs on pregnancy and childbirth, and to hypothesize strategies for improving the offered services	Grounded theory, interviews	Eight thematic areas were identified, highlighting difficulties in relationships and communication between foreign patients and medical staff. The findings revealed a common belief among midwives that maternity, childbirth, and breastfeeding follow a ‘universal grammar’, which can lead to underestimating the need for culturally competent services. Additionally, there was a contrast in participants' discourse between an egalitarian approach to service delivery and a desire for differentiated attention to diversity.
Dharni et al. (2021), UK	The key components of a successful model of midwifery‐led continuity of carer, without continuity at birth: findings from a qualitative implementation evaluation	To evaluate the implementation of a midwife‐led continuity of carer model that excluded continuity of carer at the birth, assessing its fidelity, reach, and satisfaction among midwives and women.	The evaluation was guided by the Conceptual Model for Implementation Fidelity and involved semi‐structured interviews	The evaluation highlighted successful implementation of the continuity of carer model for antenatal and postnatal care, with high levels of satisfaction reported by both women and midwives, despite the exclusion of the birth element. The positive outcomes were attributed to structural and resource factors, such as additional time and smaller caseloads, which may not be widely available within maternity unit budgets.
Shahinfar et al. (2021), Iran	Women's perception of continuity of team midwifery care in Iran: a qualitative content analysis	To explore pregnant women's perceptions of continuity of team midwifery care in Iran	Qualitative study, semi‐structured interviews	The findings emphasize the positive impact of the continuity of team midwifery model on enhancing empowerment and satisfaction among women during the perinatal period, indicating its potential for effective implementation in Iran's maternity care system.
Buchanan et al. (2022), Australia	Care ethics framework for midwifery practice: A scoping review.	To explore how ethical principles are applied among health professionals to build a definition of ‘midwifery practice’ consistent with the application of care ethics for midwifery practice	Scoping review	The use of care ethics among health professionals enhances ethical sensitivity. A proposed framework and definition for care ethics in midwifery practice are outlined, making this review relevant for midwives and other health practitioners aiming to improve their ethical sensitivity.
Muggleton and Davis (2022), Australia	Applying Salutogenesis in Midwifery Practice.	To highlight the unique aspects of midwifery as a healthcare profession that focuses on physiological processes and transitional periods in a woman's life.	Book chapter	It identifies a resonance between midwifery practice and salutogenesis, although research on their relationship is limited. The chapter suggests that aligning midwifery with salutogenic principles can enhance health promotion within midwifery practice.
MacDougall and Johnston (2022), Canada	Client experiences of expertise in midwifery care in New Brunswick, Canada.	To investigate women's perception of midwifery care in a Canadian region	Qualitative descriptive study, semi‐structured interviews	Participants expressed high satisfaction with midwifery services, highlighting factors that contributed to quality care, such as competence, adequate time and access, trauma‐informed practices, and support for autonomy. The findings indicate that midwifery care aligns with the principles of respectful maternity care. Clients viewed midwives as specialized, evidence‐based practitioners, challenging the perception of midwifery as merely traditional care in a context of underfunded reproductive healthcare.
Kızılkaya and Dolgun 2022, Turkey	A Lighthouse for Midwifery Practices: Model of Woman‐Centred Care	To examine the basic concepts of Midwifery Model of Woman‐Centred Childbirth Care and its applicability in midwifery practice.	Review	The findings suggest that MiMo could enhance midwifery practice in Sweden and potentially in other cultural contexts, including Turkey, where a woman‐centred approach is essential for improving the quality of maternity care services.
Neerland and Skalisky (2022) USA	A Qualitative Study of US Women's Perspectives on Confidence for Physiologic Birth in the Birth Center Model of Prenatal Care	to enhance understanding of the components of the US birth center model of prenatal care and how this model fosters birthing people's confidence in physiologic childbirth.	Qualitative descriptive study, semi‐structured interviews	Participants felt that the culture and environment of the birth center, the midwifery care model, their internal beliefs about birth, and external support contributed significantly to their confidence in experiencing physiologic childbirth.
Cummins et al. (2022), Australia	Exploring the value and acceptability of an antenatal and postnatal midwifery continuity of care model to women and midwives, using the Quality Maternal Newborn Care Framework	to explore the value and acceptability of an antenatal and postnatal midwifery program to women, midwives, and obstetricians prior to the implementation of the model at one hospital in Metropolitan Sydney, Australia	Qualitative descriptive study, focus groups and one‐on‐one interviews	The findings demonstrate the value and acceptability of implementing this model of care from the perspectives of women, midwives, and obstetricians.
Combellick et al. (2023), USA	Midwifery care during labor and birth in the United States.	To describe the practice of midwifery with particular emphasis on the intrapartum setting	Review	The review highlights that the U.S. has significantly fewer midwives compared to other high‐income countries, which often leads to higher maternal and neonatal mortality rates and increased healthcare costs. Midwifery care is linked to fewer medical interventions, lower cesarean rates, and greater patient satisfaction. The importance of collaboration between midwives and physicians is emphasized as essential for improving care.
Cutajar et al. (2023), Australia	Model of care matters: An integrative review	To explore women's experiences of pregnancy care accessed during pregnancy	Integrative review	Insufficient integration between medical and midwifery models contributed to dissatisfaction and distress among women during pregnancy. Positive experiences were linked to the establishment of connections with care providers. Developing a well‐informed decision aid could address information gaps and clarify the differences among various care models.
Murray‐Davis et al. (2023), Canada	Making Space for Midwifery in a Hospital: Exploring the Built Birth Environment of Canada's First Alongside Midwifery Unit	To assess the impact of the built environment of the alongside midwifery unit (AMU) on both service users and midwives	Mixed‐Method: structured online survey, interviews, and focus group	The findings indicated high satisfaction levels with the birth environment. A theoretical model was developed, highlighting that ‘making space’ for midwifery within the hospital contributed to positive birth experiences and overall satisfaction with the built environment. The core elements of this model included creating a domestic atmosphere in an institutional setting, shifting away from a technological focus, and promoting shared ownership of the unit.
Buchanan et al. (2023), Australia	Woman‐centred ethics: A feminist participatory action research	To explore women's experiences of maternity care from an ethical perspective, focusing on how they perceive ethical and unethical care in a midwifery model	Feminist Participatory Action Research, focus groups and individual in‐depth interviews	Participants described two ethical approaches to care: Woman‐centred ethics and Authoritarian ethics. The study highlighted the importance of individualized, respectful relationships and shared knowledge, with the conceptual model of *Woman‐centred ethics* proposed to improve ethical midwifery care.

### 6.2 Antecedents

Based the focus of this study, themes that describe circumstances or factors necessary for the provision of MC were identified. These antecedents include philosophy, personal features, regulatory features, context of care and professional team (Combellick et al. 2023; Murray‐Davis et al. 2023; Sharp 1998; Page and Mander 2014; Wallace 2019; Niles et al. 2021; Naughton et al. 2021; Muggleton and Davis 2022; Kızılkaya and Dolgun 2022; Neerland and Skalisky 2022; Buchanan et al. 2023; Meier Magistretti et al. 2016; Eri et al. 2020; Dettwyler et al. 2019).

The *philosophy* of care reflects an orientation that values physiology, naturalness and the respect for individual rhythms.

*Personal features* include the knowledge, skills, attitudes and values midwives bring to their practice, shaped by both education and experience.

*Regulatory features* encompass the formal frameworks that guide and constrain practice, such as policies, guidelines and professional standards.

The *context of care* refers to organisational conditions, such as time, space and environment, that support or hinder quality care.

Lastly, the *professional team* highlights the importance of collaboration, trust and role clarity among colleagues in enabling sustainable care delivery.

### 6.3 Attributes

In relation to MC, we have identified themes that enable to provide a complete description of the concept. For this purpose, they have been grouped into 2 categories: *relationship*, and family centredness (Combellick et al. 2023; Murray‐Davis et al. 2023; Sharp 1998; Page and Mander 2014; Wallace 2019; Niles et al. 2021; Naughton et al. 2021; Muggleton and Davis 2022; Kızılkaya and Dolgun 2022; Neerland and Skalisky 2022; Buchanan et al. 2023, 2022; Meier Magistretti et al. 2016; Eri et al. 2020; Dettwyler et al. 2019; Borrelli et al. 2016; Wikberg 2021; MacDougall and Johnston 2022; Byrom and Downe 2010; Hunter et al. 2016; Walsh and Devane 2012; Dharni et al. 2021; Cummins et al. 2022; Cutajar et al. 2023; Fahy and Parratt 2006; Brown et al. 2016; Newton et al. 2016; Shahinfar et al. 2021; Converso et al. 2021).

*Relationship* refers to the trusting and continuous bond developed between midwives and families. This connection is characterised by mutual engagement, emotional presence and shared decision‐making, and is supported through communication, support and advocacy throughout the care journey.

*Family centredness* complements this dynamic by ensuring that care is shaped around the family's values, preferences and specific needs. It highlights the midwife's ability to adapt, mediate and provide care that is sensitive to emotional, social, cultural and spiritual dimensions, fostering truly individualised support.

### 6.4 Consequences

Considered the focus of this study on MC, we identified themes, that describe the impact that the provision of the concept has on the population and professionals. After analysing the themes, 3 categories were identified: safety, empowerment, professional outcomes (Combellick et al. 2023; Murray‐Davis et al. 2023; Sharp 1998; Page and Mander 2014; Wallace 2019; Niles et al. 2021; Naughton et al. 2021; Muggleton and Davis 2022; Kızılkaya and Dolgun 2022; Neerland and Skalisky 2022; Buchanan et al. 2023, 2022; Meier Magistretti et al. 2016; Eri et al. 2020; Dettwyler et al. 2019; Coates and Foureur 2019; Borrelli et al. 2016; Wikberg 2021; MacDougall and Johnston 2022; Byrom and Downe 2010; Hunter et al. 2016; Walsh and Devane 2012; Dharni et al. 2021; Cummins et al. 2022; Cutajar et al. 2023; Fahy and Parratt 2006; Brown et al. 2016; Newton et al. 2016; Shahinfar et al. 2021).

*Safety* refers to the protection of families not only in clinical terms, but also in emotional, psychological, social, cultural and spiritual dimensions.

*Empowerment* captures how midwifery care fosters families' awareness, confidence and active participation in their care, enhancing autonomy and adaptability.

*Professional outcomes* concern the benefits for midwives themselves, including improved emotional well‐being, professional satisfaction and a sense of autonomy and growth within their role.

### 6.5 Surrogate Terms and Related Concepts

No different surrogated terms were identified during the analysis of the sources than those identified during the identification of the sources, namely *Maternity* and Maternity Care (Niles et al. 2021).

On the other hand, in addition to the related concepts identified during the source identification phase, namely *Sexual Health Care*, and *Reproductive Health Care* (Rodgers and Knafl 2000), during the source analysis, *Pregnancy Care* (Niles et al. 2021), *Cultural Caring* (Wikberg 2021), *Care Ethics* (Buchanan et al. 2022), *Health Promotion* (Muggleton and Davis 2022), *Salutogenesis* (Muggleton and Davis 2022), and *Post‐Partum Care* (MacDougall and Johnston 2022) were also identified.

### 6.6 Definition

Drawing on the results of this analysis, MC can be defined as a specialised care practice focused on supporting women and families through the physiological phenomena that characterise their sexual and reproductive lives (Wallace 2019) (e.g., menarche, ovarian cycle, menstrual cycle, sexuality, pregnancy, labor, delivery, postpartum period, breastfeeding, motherhood, menopause, climacteric).

MC is grounded in the philosophy of promoting natural and physiological processes prioritising the health and safety of individuals by respecting and supporting their natural rhythms and variations due to the individual subjectivity. Central to MC is the establishment of deep, continuous relationships with women and families.

All individuals, including midwives, involved in MC play an equal role in the dynamics of the individual‐ and family‐based relationship. This promotes open communication and shared decision‐making, conditions conducive to meeting the unique needs and preferences of each individual and family, promoting their autonomy and informed choice.

Moreover, MC always has as its primary concern, which is to maximise the safety of individuals and families, in its broadest view, encompassing physical, psychological, social, moral, cultural and religious aspects.

## 7 Identification of an Exemplar of the Concept

According to Rodgers's methodology, identifying an exemplar is a crucial step in a concept analysis, providing a practical illustration of the concept in a relevant context (Rodgers and Knafl 2000). In fact, understanding the concept within the environments where it occurs offers valuable insights.

In this case, we have identified an exemplar for MC based on themes identified during our analysis, which predominantly contextualise MC within organisational and institutional contexts. While the identified themes predominantly emphasise the contrasts between clinical settings and MC, the synthesis between the study findings and the themes presented in Table 5, enabled us to identify an exemplar of the concept.

**TABLE 5:** Key features of obstetric‐led models of care where midwifery care is delivered, identified in 17 sources out of 30.

Sources	Features					Conflict		Occupational health		
	Bureaucracy	Rigidity	Interventionist attitude	Fragmentation of care	‘Organization oriented’	Paradigmatic contrast	Cross‐disciplinary conflicts	Performance anxiety, frustration, burnout	Moral distress	Professional devaluation
Sharp (1998)		X		X		X	X			
Walsh and Devane (2012)	X					X				
Page and Mander (2014)		X	X				X	X		
Brown et al. (2016)		X		X	X	X				
Meier Magistretti et al. (2016)				X	X	X			X	
Newton et al. (2016)						X				X
Niles et al. (2021)		X			X	X				
Naughton et al. (2021)					X	X		X		X
Converso et al. (2021)		X								
Dharni et al. (2021)		X						X	X	
Muggleton and Davis (2022)						X				
MacDougall and Johnston (2022)	X	X				X				
Kızılkaya and Dolgun (2022)						X				
Neerland and Skalisky (2022)						X				
Cummins et al. (2022)	X	X				X		X		
Combellick et al. (2023)						X		X		
Murray‐Davis et al. (2023)						X				

A pertinent example is the implementation of midwife‐led birth centres in informal settlements, as described in Wallace's case study (Wallace 2019). These centres were established within underserved urban areas to provide accessible, respectful and culturally sensitive care to marginalised populations. The model emphasised community integration, with midwives offering continuous support throughout the antenatal, intrapartum and postnatal periods. Organisational structures were designed to be flexible, allowing midwives to exercise autonomy while ensuring timely referrals when necessary. This approach not only improved maternal and neonatal outcomes but also fostered trust within the community, demonstrating the effectiveness of midwifery care in challenging settings (Wallace 2019).

Regardless of the specific organisational context, MC is supported by flexible and adaptable structures that prioritise individual‐centred care. Rather than imposing rigid bureaucracy, protocols are designed to give midwives the autonomy to respond to specific patient needs, aligning clinical practice with holistic care principles.

Interventionist attitudes are balanced by an emphasis on natural processes, where individualised care is central, and interventions are integrated thoughtfully when necessary. Care delivery is cohesive, eliminating fragmentation through seamless coordination between departments and professionals, ensuring continuity of care.

Organisational goals and midwifery values are harmonised, fostering a supportive environment for collaborative, cross‐disciplinary relationships that minimise conflict and enhance mutual respect.

The work environment actively addresses performance pressures, promoting well‐being and reducing burnout through recognition of the midwife's role and professional value. This supportive context empowers midwives to deliver empathetic, high‐quality care, free from moral distress and professional devaluation, resulting in optimal outcomes for patients and professionals alike.

## 8 Conceptual Overview of Midwifery Care

The thematic analysis led to the identification of multiple characteristics that contribute to the conceptualisation of midwifery care. These characteristics have been organised into 5 antecedents, 2 attributes and 3 consequences, which are presented in Table 6. For each of these elements, the table also specifies the sources from which the information was drawn, offering transparency regarding the literature supporting each aspect of the concept.

**FIGURE 2:**
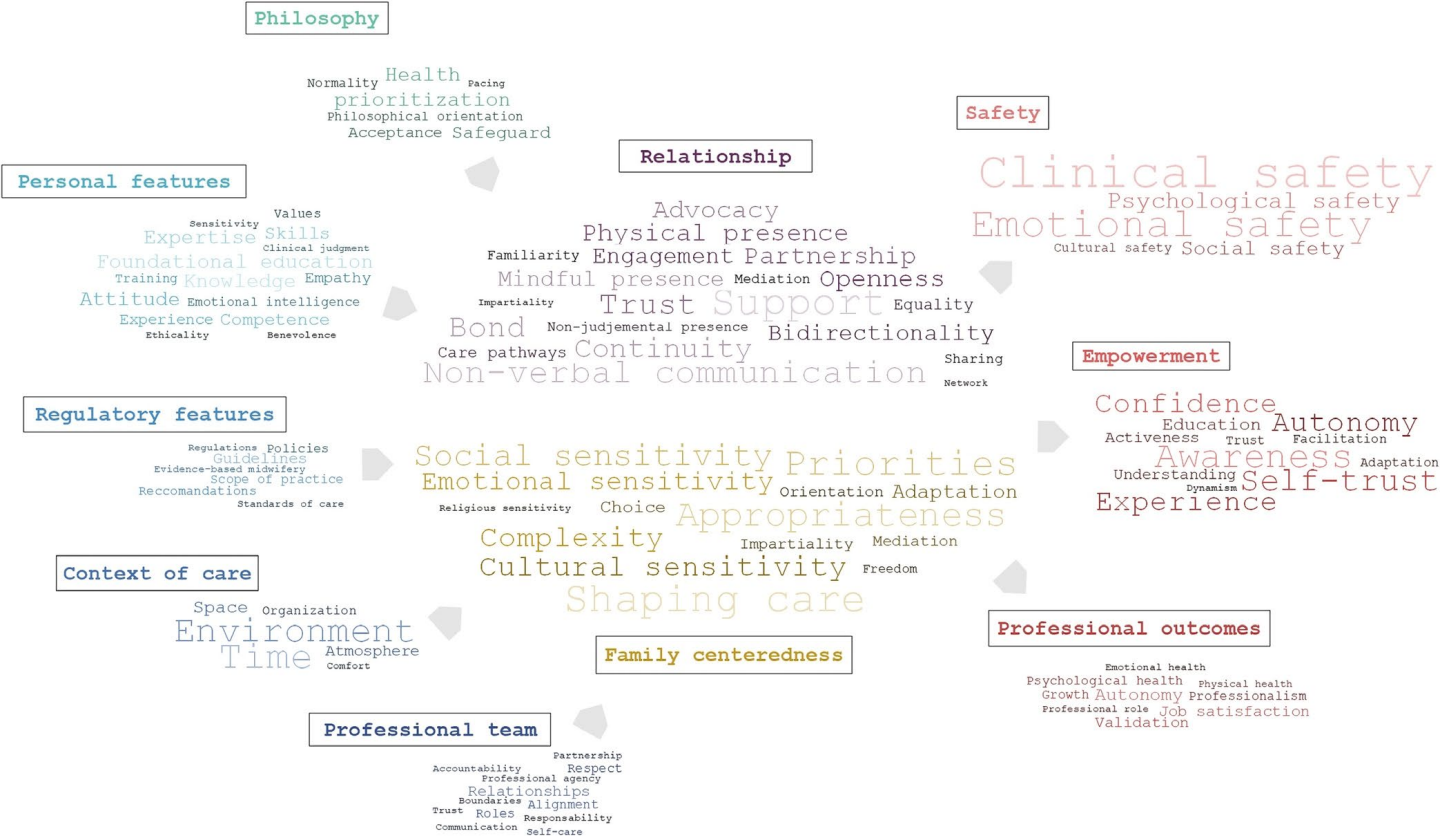
Conceptual map of keywords identified in the literature, organised from left to right into antecedents, attributes and consequences of midwifery care (font size reflects frequency in sources).

To highlight and represent the complexity and multidimensionality of the antecedents, attributes and consequences of midwifery care, we complemented the tabular presentation with a visual mapping (Figure 2). In addition to categorising the keywords, the diagram conveys their relative frequency across sources: font size was assigned in proportion to the number of sources in which each keyword appeared, with terms occurring more frequently displayed in larger font and those occurring less frequently shown in smaller font. Keywords with the same frequency share the same font size.

## 9 Discussion

### 9.1 Evolution of the Concept of Responsibility

Considering the evolutionary orientation of the concept analysis method employed (Rodgers and Knafl 2000), the findings were interpreted in light of the development and progression of the themes identified across the included sources. While most of the key elements associated with MC remain consistent and stable over time, a noteworthy evolution emerged regarding the theme of responsibility.

Initially, in the reviewed literature, the philosophy of MC acknowledged the central role of the healthcare professional in assuming responsibility for care decisions—particularly in increasingly complex care environments—while families were perceived as less prepared to navigate such settings (Sharp 1998). More recent sources, however, reveal a shift toward a more collaborative view of responsibility, emphasising shared, informed and conscious decision‐making processes between professionals and families (Wallace 2019; Meier Magistretti et al. 2016; Dettwyler et al. 2019).

This temporal development in the conceptualisation of responsibility reflects a broader trend in healthcare that encourages a move away from authoritative knowledge toward experiential knowledge. As discussed in the literature (Ketler 2000), this transition suggests a new equilibrium between different forms of knowledge within the care relationship.

By enhancing the value of experiential knowledge, this shift reconfigures traditional power dynamics in care, moving the centre of decision‐making toward women and, in parallel, midwives. These rebalancing challenges the historical dominance of biomedical discourse, supporting a more equitable and collaborative model of care that affirms the expertise of both women and midwives (Newnham 2015).

### 9.2 Influence of Care Models and Settings on Antecedents

Regarding the antecedents identified in this study, particular attention is given to the contexts in which MC is provided, with a focus on the models of care adopted. These models are strongly shaped by the clinical settings in which they operate. The philosophical underpinning is central when contrasting midwifery‐led and obstetric‐led models, which fundamentally differ in their guiding care philosophies (Bryar 1988), often explaining the contrasts highlighted in Table 5.

**TABLE 6:** Overview of the antecedents, attributes, and consequences of midwifery care identified in the included sources.

Source	Antecedents					Attributes		Consequences		
	Philosophy	Personal features	Regulatory features	Context of care	Professional team	Relationship	Family centredness	Safety	Empowerment	Professional outcomes
Sharp (1998)	X	X	X	X		X	X	X	X	
Fahy and Parratt (2006)				X		X		X	X	
Byrom and Downe (2010)		X				X		X		
Walsh and Devane (2012)		X		X	X	X		X	X	
Page and Mander (2014)	X	X		X		X	X	X		
Brown et al. (2016)		X		X		X	X	X		X
Meier Magistretti et al. (2016)	X			X		X	X	X	X	X
Hunter et al. (2016)		X		X	X	X		X		X
Borrelli et al. (2016)		X	X	X	X	X	X	X	X	
Newton et al. (2016)				X		X	X	X	X	X
Wallace (2019)	X	X	X	X		X	X	X		
Dettwyler et al. (2019)	X	X	X		X	X	X		X	
Coates and Foureur (2019)		X						X		
Eri et al. (2020)	X	X		X		X	X		X	
Niles et al. (2021)	X	X		X		X	X	X	X	X
Wikberg (2021)		X				X	X	X	X	
Naughton et al. (2021)	X					X	X	X	X	
Converso et al. (2021)						X	X			
Dharni et al. (2021)				X		X	X	X	X	X
Shahinfar et al. (2021)						X	X		X	
Buchanan et al. (2022)						X	X	X	X	
Muggleton and Davis (2022)	X	X						X	X	
MacDougall and Johnston (2022)		X	X	X		X	X	X	X	
Kızılkaya and Dolgun (2022)	X	X	X	X		X	X	X	X	X
Neerland and Skalisky (2022)	X	X		X	X	X	X	X	X	
Cummins et al. (2022)				X		X	X	X	X	
Combellick et al. (2023)	X	X	X	X	X	X	X	X	X	
Cutajar et al. (2023)				X		X	X	X	X	
Murray‐Davis et al. (2023)	X	X								X
Buchanan et al. (2023)	X	X				X	X		X	

In settings dominated by obstetric‐led models, essential ‘contextual antecedents’ such as time (Niles et al. 2021; Dharni et al. 2021; Brown et al. 2016), space and atmosphere (Neerland and Skalisky 2022) are frequently lacking. These factors have been shown to influence physiological processes like labor and birth, not only through neurological and hormonal pathways (Hammond et al. 2013) but also from the perspective of care ethics (Gallagher 2020), underscoring their importance in delivering high‐quality MC.

Such challenges are particularly evident in large hospital‐based maternity wards, where high patient turnover, standardised protocols and staff shortages often limit the feasibility of sustained MC (MacDougall and Johnston 2022; Cummins et al. 2022).

Furthermore, obstetric‐led models tend to prioritise organisational requirements, resulting in regulatory frameworks that may undervalue MC's potential (Naughton et al. 2021; Meier Magistretti et al. 2016; Cummins et al. 2022; Converso et al. 2021) and thus impede its effective implementation.

### 9.3 Ethical Implications of Organisational Models

From an organisational perspective, this conflict raises a significant ethical concern. All women and families, regardless of the care model they choose or their clinical risk levels, have an inherent right to high‐quality MC whenever appropriate (ICM 2024). Therefore, the absence of essential antecedents for MC within obstetric‐led models results in systemic discrimination by limiting equitable access to MC and its associated benefits. Women and families in such settings face a greater risk of being denied care that acknowledges and respects their individual needs, preferences and experiences, which is a core element of ethical care practice (Rayment‐Jones et al. 2023).

This ethical concern is particularly evident in settings where care is organised around institutional priorities rather than the needs of women. In contrast, models rooted in MC, such as those implemented in most freestanding birth centre or midwifery units, demonstrate how ethical principles, like respect for autonomy, beneficence and justice, can be operationalised through organisational structures (MacDougall and Johnston 2022; Cutajar et al. 2023).

Furthermore, this conflict extends to the professionals working within these environments. The personal attributes identified as characteristic of midwives often conflict with the contextual features described above (Combellick et al. 2023; Niles et al. 2021; Naughton et al. 2021; Meier Magistretti et al. 2016; MacDougall and Johnston 2022; Brown et al. 2016) and are frequently unrecognised or undervalued by other healthcare professionals interacting with midwives in these settings (Page and Mander 2014; Cummins et al. 2022).

This issue reflects a broader complex dynamic between professional groups, particularly between midwives and obstetricians, which influences interprofessional interactions within care settings (Reiger 2008). As a result, midwives frequently report strong ethical and professional commitment to delivering high‐quality MC; however, structural limitations inherent to obstetric‐led models impede their ability to uphold this commitment (Foster et al. 2021), often leading to professional dissatisfaction and moral distress (Foster et al. 2021; Suleiman‐Martos et al. 2020).

### 9.4 Central Attributes: Relationships and Family Centredness

Considering the attributes identified in the study, the importance of relationships and the centrality of the woman are well supported by existing literature (Bradfield et al. 2019; Brady et al. 2024; de Jonge et al. 2021; Rocca‐Ihenacho et al. 2021). However, based on the themes identified in the sources, the use of the term ‘family centredness’ was deemed appropriate, given the recognised need, when providing MC, to also engage with the woman's support system and the network of care identified by the woman herself (Sharp 1998; Wallace 2019; Wikberg 2021; MacDougall and Johnston 2022; Newton et al. 2016). Furthermore, identifying these attributes within a single analysis has highlighted their complementarity, as one cannot exist without the enhancement of the other, and vice versa.

The consequences of MC, as revealed by the analysed sources, are strongly supported by extensive literature confirming the relationship between MC and midwifery‐led models of care and improved outcomes, both in the short (Yu et al. 2020; Homer et al. 2019) and long term (Watson et al. 2021; Keedle et al. 2023). These effects, encompassing both physical and psychological outcomes, are observed not only in women and newborns, but also extend to families, communities (McNeill et al. 2012), and professionals (Hanley et al. 2022; Hunter et al. 2016).

### 9.5 Related Concepts and Fragmentation of Care

It is pertinent to propose a categorisation of the related concepts identified in the sources into two distinct groups. The first group comprises concepts such as Salutogenesis, Cultural Caring and Care Ethics, which address fundamental philosophical dimensions relevant to the concept under analysis. The second group includes Sexual Health Care, Reproductive Health Care, Pregnancy Care, Health Promotion and Post‐Partum Care.

These terms illustrate how midwifery‐related phenomena, representing a continuum of care throughout an individual's life, are frequently fragmented in clinical practice regarding intake and management, as supported by findings from this study (Table 5) and previous literature (Tracy et al. 2013). Indeed, the concepts within the second group may be interpreted as fragments of the broader MC concept, each concentrating on a specific phase within the continuum of MC.

## 10 Strengths and Limitations

In general, the findings of this concept analysis are well‐supported by the existing literature. Nevertheless, the distinctive strength of this study lies in the methodological approach adopted. By focusing specifically on MC, this methodology allows for a systematic and thorough examination of the fundamental aspects that characterise MC.

This approach not only underscores each component of MC but also captures a broader perspective of the contexts where it is delivered, offering a more comprehensive and holistic understanding of the processes both before and after its provision. Consequently, the study contributes to a clearer definition of the concept, enhancing its relevance in both professional practice and care settings.

Importantly, this document addresses gaps previously unexamined, and its strength lies in its potential for use in both undergraduate and postgraduate education, providing a valuable resource for future midwifery training.

Despite these strengths, some limitations must be acknowledged. One of the key limitations relates to the sources included in the literature review. Nearly all the studies were conducted in high‐income countries, which may limit the applicability of the findings in lower‐income countries and their healthcare settings and contexts.

Furthermore, although the sources provided robust data to address the objectives of this study, they were thematically homogeneous. This thematic narrowness limited the exploration of contemporary issues, particularly those that intersect with the principles of justice, equity, diversity and inclusion (JEDI). As such, most of the studies do not directly engage with the role of MC and midwives in addressing the health needs of marginalised groups, including minorities, disadvantaged populations and members of the LGBTQ+ community.

Considering these findings and the limitations noted, this study can serve as a foundation for future research aimed at exploring how the insights identified here may apply in other contexts, such as middle‐ and low‐income countries or among specific populations with unique characteristics, thereby allowing the conceptualisation of midwifery care to evolve and adapt to diverse healthcare settings and populations.

## 11 Conclusion

Given the adaptive nature of concepts, the understanding of MC may continue to evolve alongside changes in healthcare contexts and societal needs. The fluidity of the concept allows it to be adapted to emerging challenges and opportunities within clinical, educational, research and organisational settings.

While the present analysis has provided a thorough foundation in defining and understanding MC, further research is needed to explore how the concept will continue to develop, particularly in response to evolving demands in healthcare, inclusiveness and interdisciplinary collaboration.

This study not only contributes to current knowledge but also lays the groundwork for an ongoing inquiry into the dynamic nature of MC and its role in advancing the profession.

## Author Contributions

**Martina Barbieri:** conceptualisation, methodology, formal analysis, investigation, data curation, writing – original draft, project administration. **Andrea Moro:** formal analysis, investigation, data curation. **Gianluca Catania:** conceptualisation, methodology, writing – review and editing, supervision, project administration. **Franco A. Carnevale:** conceptualisation, methodology, writing – review and editing, supervision, project administration. **Giuseppe Aleo:** supervision. **Milko Zanini:** supervision. **Loredana Sasso:** supervision. **Annamaria Bagnasco:** conceptualisation, methodology, supervision, project administration.

## Ethics Statement

This study did not require ethical approval. All the phases of the research were conducted in accordance with established principles of research ethics. The exemplar was identified based on themes identified in the included sources, without the need for additional materials.

## Consent

The authors have nothing to report.

## Conflicts of Interest

The authors declare no conflicts of interest.

## Supporting information


**Data S1:** nop270354‐sup‐0001‐Supinfo1.pdf.

## Data Availability

Data available on request from the authors: The data that support the findings of this study are available from the corresponding author upon reasonable request.
